# Drug transport by red blood cells

**DOI:** 10.3389/fphys.2023.1308632

**Published:** 2023-12-11

**Authors:** Sara Biagiotti, Elena Perla, Mauro Magnani

**Affiliations:** Department of Biomolecular Sciences, University of Urbino, Urbino, Italy

**Keywords:** red blood cells, drug transport, blood-to-plasma ratio, pharmacokinetic, blood distribution

## Abstract

This review focuses on the role of human red blood cells (RBCs) as drug carriers. First, a general introduction about RBC physiology is provided, followed by the presentation of several cases in which RBCs act as natural carriers of drugs. This is due to the presence of several binding sites within the same RBCs and is regulated by the diffusion of selected compounds through the RBC membrane and by the presence of influx and efflux transporters. The balance between the influx/efflux and the affinity for these binding sites will finally affect drug partitioning. Thereafter, a brief mention of the pharmacokinetic profile of drugs with such a partitioning is given. Finally, some examples in which these natural features of human RBCs can be further exploited to engineer RBCs by the encapsulation of drugs, metabolites, or target proteins are reported. For instance, metabolic pathways can be powered by increasing key metabolites (i.e., 2,3-bisphosphoglycerate) that affect oxygen release potentially useful in transfusion medicine. On the other hand, the RBC pre-loading of recombinant immunophilins permits increasing the binding and transport of immunosuppressive drugs. In conclusion, RBCs are natural carriers for different kinds of metabolites and several drugs. However, they can be opportunely further modified to optimize and improve their ability to perform as drug vehicles.

## 1 The unique properties of human red blood cells that have an impact on drug transport

Human red blood cells (RBCs) represent 99% of the cellular compartment in the blood and comprise the most numerous cells in the body. One microliter of blood contains approximately 4–5 million RBCs, which means approximately 25 million million RBCs in the total body of an adult human being. Moreover, thanks to their shape and deformability, they can reach almost all organs and tissues and, for this reason, they are considered the carriers par excellence. Mature RBCs mainly contain hemoglobin; thus, it can be argued that RBCs are passive carriers and that their role only relies on oxygen and CO_2_ transport (Hsia, 1998). Indeed, a lot of other molecules can be carried by RBCs. As a matter of fact, erythrocytes are not mere gas transporters, but they are also involved in other functions, i.e., vascular function, coagulant pathways, defense processes, and metabolic pathways, thanks to their further contents (Helms et al., 2018; Olver, 2020; Stosik et al., 2020). Regarding vascular function, they are the main source of nitric oxide, and consequently, they are involved in blood pressure homeostasis as well. Concerning metabolism involvement, physiological examples are given by amino acids, in particular the alanine’s transport in the so-called glucose–alanine cycle through the muscle tissue and liver. It was demonstrated that the carriage percentage of alanine is higher in whole blood, thanks to the binding with RBCs, than in plasma (Felig et al., 1973). Other examples are nucleosides which are major precursors for nucleotide biosynthesis. RBCs present different specific membrane transport systems for nucleosides, including the equilibrative nucleoside transporter 1 (ENT1), whose absence is related to defective erythropoiesis (Mikdar et al., 2021). Finally, RBCs are also important carriers of hormones, folate, drugs (e.g., valproate, phenytoin, and hydrocortisone), and small metabolites that play key roles in gas exchanges (Highley and De Brujin, 1996). All of these molecules are involved in a complex homeostatic balance between in- and out-flux (Kirk, 2001; Sharma et al., 2001) mediated not only by passive diffusion but also by the use of active transport mechanisms (Sharma et al., 2001), which may be involved in the transport of drugs.

## 2 RBCs as natural carriers for drugs

**FIGURE 1:**
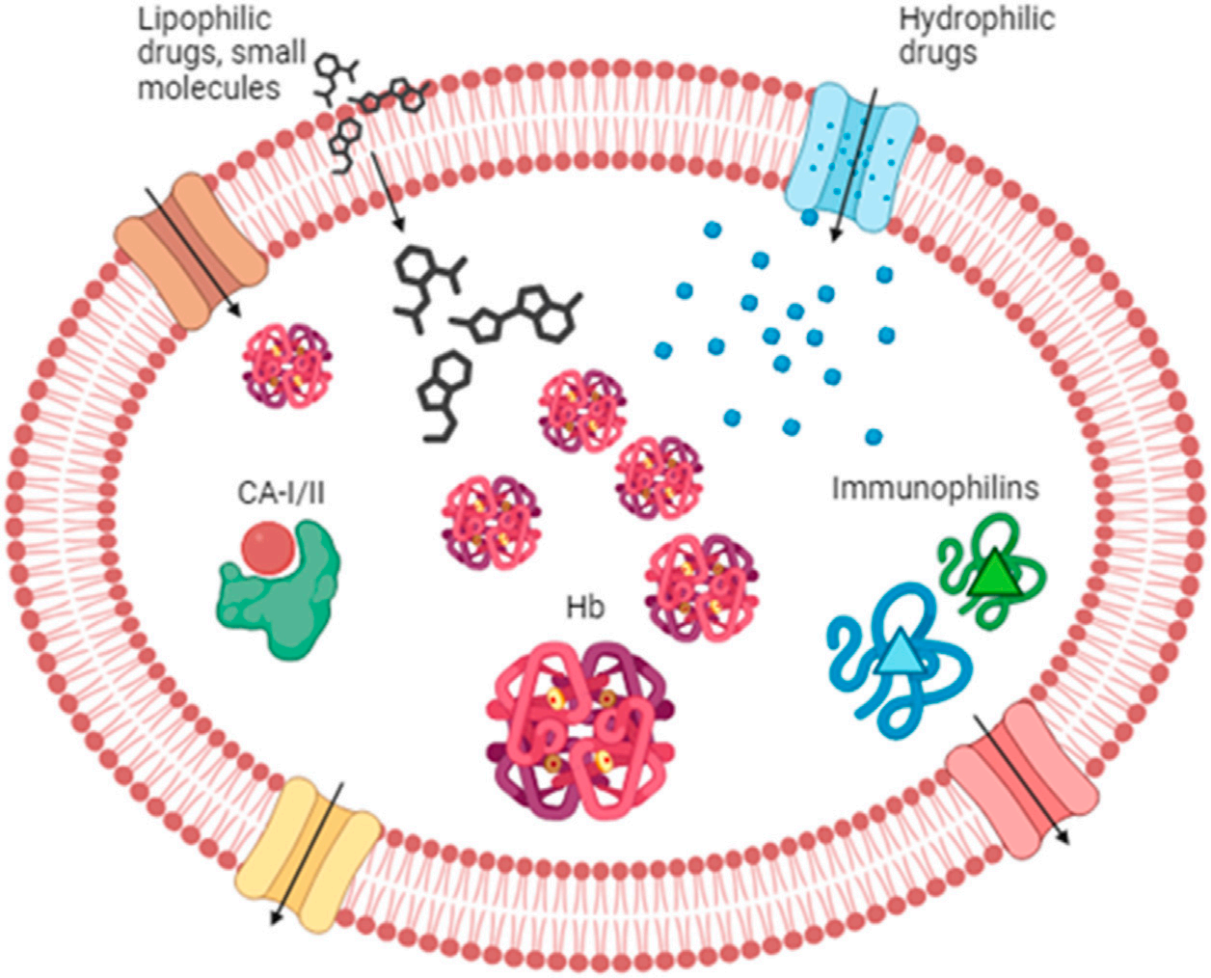
Drug partitioning into RBCs. Lipophilic drugs and small molecules can enter the RBC through the lipid bilayer, while hydrophilic compounds can enter via the aqueous channels (in blue) or other transporters such as Glut 1 (in brown). Once in the cytoplasm, drugs can find several binding sites. Among those, we can find hemoglobin, carbonic anhydrase, and immunophilins that possess an affinity for several drugs. RBC partitioning is also affected by drug efflux across several transporters like P-glycoprotein and multidrug resistance-associated protein (MRPs) (in red and yellow, respectively). Hb, hemoglobin; CA-I/II, carbonic anhydrase I and II.

The RBC surface area is approximately 163 μm^2^ (Beutler et al., 1995), and with a few calculations, we can estimate a total surface area of more than 4,000 m^2^. In light of the above, it is quite easy to understand that with a similarly wide area, drugs can easily be distributed into the RBC compartment. In particular, drug molecules can bind to the membrane and/or to proteins into the cytosol. Among the drugs that can bind to the RBC membrane, we can cite codeine, mefloquine, chlorpromazine, imipramine, pyrimethamine (Hinderling, 1997; Dash et al., 2021), and several others. As a matter of fact, the RBC membrane provides an extended surface area that may also be used for anchoring various therapeutic molecules intended to act in the bloodstream. Interested readers can find more information in papers published by Murciano et al. (2009), Muzykantov (2010), Carnemolla et al. (2017), Villa et al. (2018), and Rossi et al. (2019). However, this review will not focus on drugs binding to the RBC membrane but on those that enter into the cell and bind to cytosolic components of RBCs. Indeed, several features affect the partitioning of drugs into RBCs, such as lipophilicity and molecular size. Lipophilic drugs can cross the RBC lipid membrane by simple diffusion, while hydrophilic compounds can enter due to the aqueous channels or other membrane-facilitated or active transport systems such as Glut1 (Dash et al., 2021). Once into the RBC cytoplasm, drugs can find several enzymes and/or proteins to bind to. The most important protein is notably represented by hemoglobin. Hemoglobin represents 10% of the total body proteins of an adult and is able to carry many substances. A review from Hinderling summarized in a table the drugs known to be bound by hemoglobin, and among them, we can find barbiturates, digoxin and derivatives, and salicylic acid (Hinderling, 1997). A more recent review also cites sulfonamides, phenytoin, phenothiazines, phenylbutazones and derivatives, imipramines and derivatives, proquazone, and pyrimethamine (Dash et al., 2021). Some of these drugs may induce allosteric modifications in the hemoglobin structure, changing its affinity for oxygen. In addition to hemoglobin, there are other proteins known to be the binding site for drugs. The nucleoside transporter can bind to draflazine and acetazolamide, while carbonic anhydrase represents the binding site for antidiuretics like chlortalidone, dorzolamide, and methazolamide (Hinderling, 1997). However, the most important example of drug-binding protein is represented by immunophilins (e.g., cyclophilin and FKB12) that bind with very high-affinity immunosuppressive drugs such as cyclosporine and tacrolimus (Magnani et al., 2012). Finally, drug partitioning into RBCs is affected by the presence of efflux transporters onto the plasma membrane. Evidence showed the presence of P-glycoprotein and breast cancer resistance protein (BCRP) in the RBC membrane. On the contrary, multidrug resistance-associated proteins MRP1, MPR4, and MPR5 are not only involved in the uptake of antimalarial drugs but also in the efflux of some metabolites (e.g., oxidized glutathione conjugates and cyclic nucleotides) (Dash et al., 2021) (Figure 1). Moreover, RBCs can be engineered as drug delivery systems, thanks to their features of biocompatibility, biodegradability, and long-circulating life (Villa et al., 2017). Recently, various types of drug delivery systems based on blood cells have been developed, as well as blood-cell-inspired carriers that mimic the features of native blood cells (Wang et al., 2022). Finally, a new kind of next-generation carrier arising from RBCs has been proposed; this is the case of RBC-derived extracellular vesicles (RBCEVs), which are promising nanosized drug carriers for the intracellular delivery of cargoes (Biagiotti et al., 2023).

**FIGURE 2:**
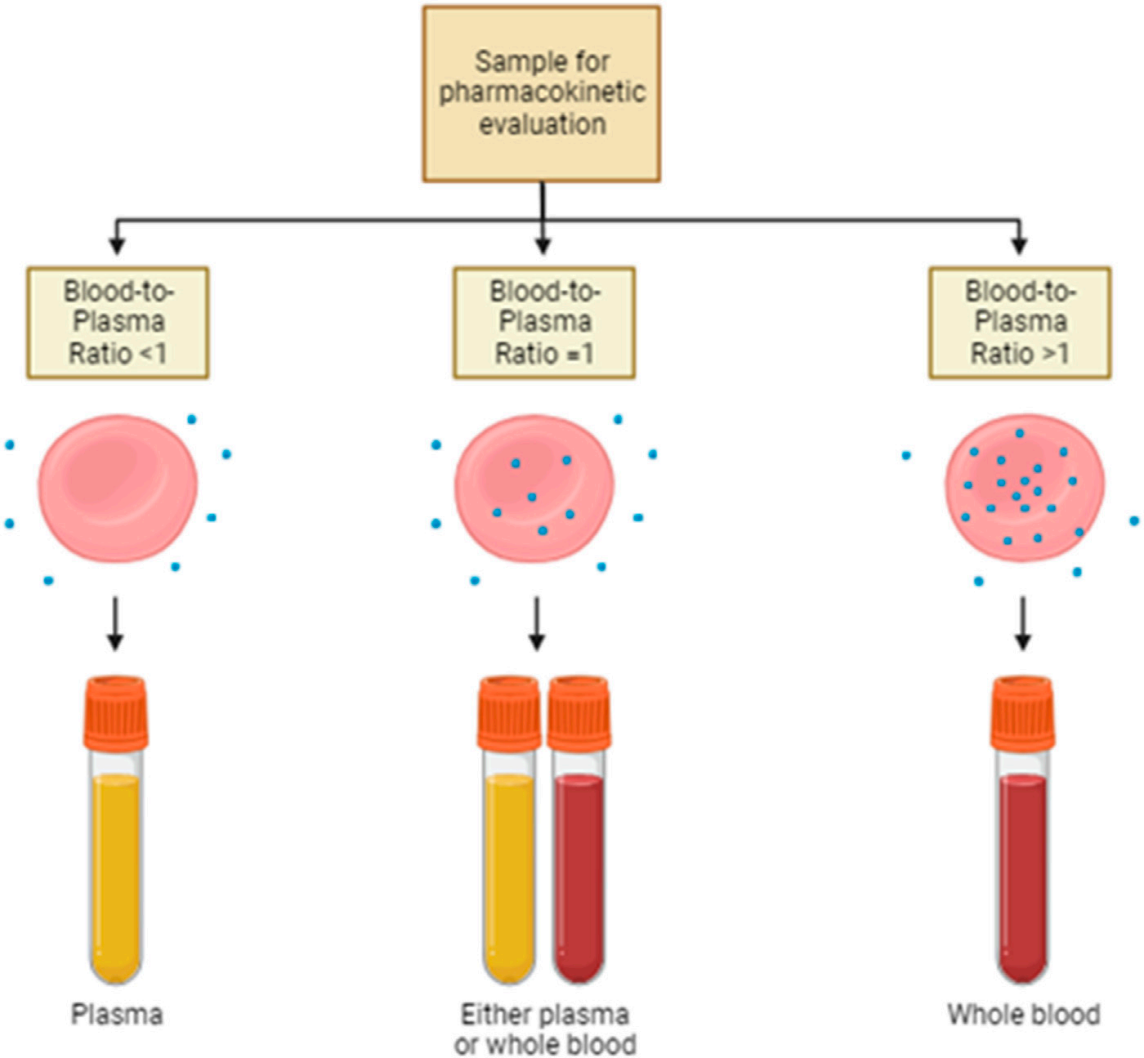
Sample choice for pharmacokinetic evaluation. Drug distribution strictly affects the pharmacokinetic properties of the same therapeutic agent. Thus, the choice of the most suitable sample for therapeutic drug monitoring is pivotal. In the case of drugs with a blood-to-plasma ratio lesser than 1, plasma is the sample of choice for pharmacokinetic evaluations. On the contrary, when the blood-to-plasma ratio is higher than 1, whole blood is the recommended sample on which the assessment of drug concentration is performed. Either plasma or whole blood is appropriate if the ratio is around 1.

Despite the big importance that has been given to plasma protein-binding drugs, the study of RBC partitioning has received much less attention over time. Currently, the routine practice focuses on the systematic investigation of the distribution of drugs into plasma or serum, while it quite completely neglected the measure of the drug distribution into RBCs. Hinderling and other authors have proposed several methods for the measurement of drug partitioning *in vitro* and/or *ex vivo* (Hinderling, 1997). This is very important, especially for the drugs that show high blood-to-plasma ratios. In other words, the plasma concentration of this drug is not at all representative of the real drug bioavailability and pharmacokinetics (Figure 2). In the following paragraphs, some examples are provided.

The drug partitioning rate between RBCs and plasma is calculated using the erythrocyte suspension in plasma and/or buffer after the times required to reach the equilibrium between the compartments and centrifugal separation of the phases. The RBC partitioning rate is calculated by measuring the ratio between the concentrations of the drug in the RBC compartment and those in the plasma or buffer. The partitioning rate in buffer can be considered a measure of the drug's absolute affinity for the RBC-binding sites, while the partitioning rate in plasma indicates the relative drug’s affinity for RBC-binding sites with respect to that of the plasma, such as albumin (Hinderling, 1997). Hence, only the unbound fraction in plasma (*i.e.,* drug molecules that are not bound to albumin or other plasma-binding sites) can partition into RBCs. However, these plasma proteins are saturable; thus, the exceeding drugs’ molecules remain unbound in plasma and can additionally partition into RBCs, apparently increasing their affinity for the erythrocyte compartment (Hinderling, 1997). Of note, RBC-associated drugs have a longer life span in circulation compared to the ones portioned in plasma because they are protected by RBCs from macrophage uptake, the liver metabolism, and renal clearance. On the other hand, engineering RBC membranes may induce some feature changes such as an increase rigidity, increasing mechanical stress-induced hemolysis, C-reactive protein activation, and phosphatidylserine (PS) translocation. All these kinds of modifications can lead to precocious clearance of RBCs from the bloodstream if not adequately treated. Several authors have investigated *in vitro* and *in vivo* biocompatibility of membrane-bound molecules, and by appropriate approaches, they have identified the best conditions that do not affect cell clearance (Ferguson et al., 2022) or permit to direct the cargo to the preferred target tissue (Villa et al., 2017; Pan et al., 2018; Glassman et al., 2022). It is worth noting that RBCs can bind to not only drugs but also immune complexes, components of the complement system, biomolecules, small thrombi, and microbial agents, affecting their clearance and eventual transfer to phagocytic cells. In our case, we consider drugs loaded within the inner volume of RBCs, which determine some morphological modifications but do not significantly affect the *in vivo* longevity of the RBC, so they are considered such an optimal drug delivery system able to avoid the liver and spleen metabolisms (Robert et al., 2022). Hence, these drugs can be slowly released into a patient’s circulation over a longer period of time (Glassman et al., 2020; 2022). Moreover, RBCs, as drug delivery systems, not only ameliorate the pharmacokinetics drugs’ profile but may also represent a new and safe means to deliver therapeutic molecules that otherwise would induce a toxic response (Zaitsev et al., 2006; 2012; Villa et al., 2018).

### 2.1 Which kind of drugs can bind to RBCs?

In the last years, several drugs have been demonstrated to be able to cross the RBC membrane and highly distribute within them. Thus, RBC partitioning of drugs should be considered at least for these drugs with known RBC affinity. Indeed, the pharmacokinetic profile of those molecules can be extremely affected by this partitioning, and thus, the blood-to-plasma ratio could be opportunely assessed.

#### 2.1.1 Immunosuppressive drugs

Since its first use in the late 80s, cyclosporine A (CsA) has revolutionized the world of transplantation by enabling patients to reduce the possibility of rejection. However, CsA exhibits significant intra- and inter-patient pharmacokinetic variabilities and has limited bioavailability. The tolerability profile of CsA is marked by several potentially severe adverse effects, i.e., acute or chronic nephrotoxicity, hypertension, and neurotoxicity. The primary dose-limiting adverse effect associated with CsA is nephrotoxicity, typically manifested as a reversible decrease in the glomerular filtration rate (Shen et al., 2010). In comparison to CsA, tacrolimus offers greater potency and a more favorable side-effect profile, leading to increased long-term survival in patients (Yang, 2010). Despite its therapeutic efficacy, tacrolimus has a narrow therapeutic window (5–20 ng/mL whole blood 10–12 h post-dose) and frequently displays episodes of toxicity, such as nephrotoxicity, neurotoxicity, and glucose intolerance (Chow et al., 1997). For this reason, therapeutic drug monitoring is highly recommended, and whole blood concentration has been selected as the gold standard pharmacokinetic parameter. As mentioned, immunosuppressive drugs, after their administration in the clinical practice, show the highest blood-to-plasma ratio, owing to an extensive spontaneous binding to RBCs. It is worth noting that these immunophilins are highly expressed in native RBCs, and this confers, to the respective drug, high RBC partitioning. Indeed, more than 70% of CsA binds to erythrocytes at concentrations ranging from 50 to 1,000 ng/mL. The cytosolic form of CsA is specifically bound to the erythrocyte peptidyl–prolyl cis–trans isomerase cyclophilin A (Malaekeh-Nikouei and Davies, 2009). The overall binding capacity of CsA to red blood cells (RBCs) is approximately 43 × 10^5^ nmol per 10^6^ RBCs (Malaekeh-Nikouei and Davies, 2009; Sander and Holm, 2009) equivalent to 2 µg CsA/mL in a suspension of RBCs at 40% hematocrit. Notably, FK506 exhibits even higher partitioning into RBCs. In blood, approximately 85% of FK506 associates with erythrocytes, followed by plasma (14%) and lymphocytes (0.46%) (Kim et al., 2008). This elevated RBC fraction is attributed to the presence of at least two types of immunophilins in erythrocytes, binding the drug with really high affinity. Immunophilins are a highly conserved family of proteins with a *cis–trans* peptidyl–prolyl isomerase activity sharing the ability to bind immunosuppressive drugs (Corcoran, 2009). Cyclophilin A was originally discovered as a specific CsA-binding protein, while FK506-binding proteins (FKBPs) as tacrolimus, also known as FK506-binding proteins (Czogalla, 2009; Fukata et al., 2011). In particular, FKBP12 is a 12-kDa cytosolic protein with peptidyl–prolyl *cis–trans* isomerase activity, while FKBP-13 is a 13-kDa membrane-associated protein with 43% amino acid identity with FKBP12 (Lai et al., 2010). A saturable binding capacity has also been calculated for FK506 and amounted to 440 ng/mL of blood (Kim et al., 2008). Furthermore, similar to CsA, FK506 exhibits considerable variability in its pharmacokinetic profile among patients. Consequently, the pharmacokinetics of immunosuppressive drugs heavily relies on their interaction with red cell immunophilins. Additionally, the tolerability of these drugs is influenced by the fraction that is unbound in plasma as the portion transported by red cells does not induce toxic side effects.

Another class of immunosuppressant is represented by the serine/threonine kinase inhibitor; they do not inhibit calcineurin but act by inhibiting mTOR kinase (Mika and Stepnowski, 2016). Sirolimus (rapamycin) is the most representative of this class; chemically, it is a 31-membered macrolide that competes with FK-506 due to the great similarity of their chemical structure. Identically to FK-506, it forms a complex with the cytosolic protein FKBP. However, it establishes a ternary complex involving the pivotal serine/threonine kinase mTOR (mammalian target of rapamycin). Similar to FK-506, sirolimus has a high affinity for erythrocytes, thanks to the presence of high FKBP expression; RBC partitioning reaches 95% of the whole drug concentration (Yatscoff et al., 1995). Hence, the establishment of therapeutic monitoring protocols for the drug is mandatory, and whole blood is the preferred matrix (Figure 2).

#### 2.1.2 Chemotherapeutics

Chemotherapeutic agents undergo oral and parenteral administration and are carried to various tissues via the bloodstream, partitioning into plasma water, plasma proteins, or cells. Red blood cells may be involved in anthracyclines, ifosfamide and its metabolites, topoisomerase I and I/II inhibitor storage, transport, and metabolism. RBCs vehicle these drug molecules to the tumor tissues and release them through different active or passive transport mechanisms. For example, among these classes of anti-tumor drugs that can spontaneously partition into RBCs, there are doxorubicin and daunorubicin. Studies both in patients and rats demonstrated that almost 50% of doxorubicin can be found in the RBC compartment after intravenous administration (Colombo et al., 1981; De Flora et al., 1986). Moreover, when increasing the administered drug dose, the RBC-bound doxorubicin concentration increases, suggesting that their storage capacity is even higher. After partitioning, this drug is released by the RLIP76 transporter, a carrier specialized in the cytotoxic agents’ elimination (Schrijvers, 2003). In addition, daunorubicin, another anthracycline drug used in the treatment of solid and liquid cancers, can be found together with its metabolites in RBCs after intravenous administration (Lachâtre et al., 2000). Lastly, the erythrocyte importance in the mercaptopurine transport and metabolism has been demonstrated. Indeed, the mercaptopurine concentration within RBCs has been proven to have a prognostic value in the treatment of childhood acute lymphoblastic leukemia (Schrijvers, 2003). These native features have been widely exploited for drug engineering and will be further analyzed in the following paragraphs.

#### 2.1.3 Metformin

Metformin, an FDA-approved antidiabetic agent, is broadly used every day for the first-line treatment of type 2 diabetes mellitus, even if its therapeutic action is still not well-defined. Remarkably, metformin demonstrated immunomodulatory features in pathological contexts, including cancer, hyper-inflammatory diseases, and infectious disease, and its potential use for other medical conditions is currently being investigated in many clinical trials (Foretz et al., 2023). Recently, its role in hematological tumors has been evaluated, showing that metformin action in lymphomas can be related to glucose metabolism (Bagaloni et al., 2021). Considering drug partitioning, metformin shows a high blood-to-plasma ratio (>10 at C min) in humans; thus, erythrocytes are considered a key distributional compartment for this drug molecule. However, the literature is currently lacking in metformin’s intrinsic *in vitro* B/*p* values. Xie F. et al. emphasized on different drug partitioning blood cells in a dynamic *in vivo* system and static *in vitro* values, especially when repartitioning from blood cells is slower than drug clearance in plasma (Xie et al., 2015). These data reveal that the clinical terminal half-life of metformin in plasma (6 h) is three- to four-fold shorter than its half-life in whole blood (18 h) and RBCs (23 h). Despite this, more demonstrative results are still needed.

#### 2.1.4 Berberine

Berberine (BBR) is a naturally occurring benzylisoquinoline alkaloid widely used in traditional Chinese medicine (Ai et al., 2021) and characterized by low concentration and high tissue distribution. Unfortunately, most pharmacokinetic research has focused on the drug’s concentration in plasma, making a deep knowledge of its pharmacokinetic process difficult. Yu K. et al. investigated how berberine interacts with RBCs and how it is combined with hemoglobin (Yu et al., 2022). The results indicated that berberine was more concentrated in RBCs than in plasma and the maximum concentration of drug loading was 0.185 g/mL, while higher concentrations have been associated with hemolysis. Actually, co-incubation of berberine and RBCs induced the internalization of the RBC membrane and intracellular vacuole development (Yu et al., 2022). Both *in vitro* and *in silico* findings showed that berberine had a binding affinity for bovine Hb and altered the molecular environment of Hb residues, such as tryptophan and tyrosine, changing the conformation of its secondary and tertiary structures. Molecular docking demonstrated a hydrogen bond-based interaction between berberine and Arg-141 residue, whereas molecular dynamics simulation proposed the establishment of a stable conformation (Wang et al., 2007). Considering all these data together, it is reasonable to assume the existence of an RBC-Hb self-assembly system and a spontaneous system of drug release by RBCs after oral or intravenous administration of this drug, which provided a new understanding of the discrepancy between the high tissue concentration and extremely low plasma concentration of berberine.

#### 2.1.5 Benzodiazepines

Benzodiazepines are among the new psychoactive substances (NPS) that are recently emerging in large numbers. To completely understand their potential benefits and side effects, information about their pharmacological features is necessary. One parameter which is still undescribed is the B/P ratio. Manchester et al. (2022) used gas chromatography tandem to mass spectrometry to determine the blood-to-plasma ratio for the following benzodiazepines: deschloroetizolam, diclazepam, etizolam, meclonazepam, phenazepam, and pyrazolam (Manchester et al., 2022). The results reported various ranges of blood-to-plasma ratios, some of which significantly differ from those found in the literature. As a result, it is incorrect to presume that blood-to-plasma ratios are always assumed to be 1.0 or equivalent to those of other species when they are unknown; instead, they must be determined. It is necessary to determine pharmacodynamics, i.e., the binding affinity to the GABA receptor, as well.

#### 2.1.6 Topiramate

Topiramate is an antiepileptic drug that acts through the inhibition of isoenzymes of carbonic anhydrase (CA). CA-I and CA-II are significantly present in RBCs and, for this reason, may influence the drug pharmacokinetics. Shank RP et al. demonstrated the topiramate linearity of the pharmacokinetics in plasma, while its clearance from whole blood seemed to increase with escalating doses (Shank et al., 2005). In contrast, as the topiramate concentration increased, the B/P ratio decreased from 8 to 2, suggesting a significant and saturable topiramate-RBC binding. Additionally, the kinetic parameter analysis indicated that the overall binding of topiramate could be due to RBC CA-I and CA-II. Consequently, RBCs may function as a reservoir for topiramate, mitigating its loss through clearance from plasma.

## 3 How we can take advantage in converting RBCs into a drug carrier or improving the delivery of physiological molecules

As is often the case, engineering mimics certain processes that occur spontaneously in nature. For example, many of the cases reported above showed the spontaneous binding of drugs to RBC-binding proteins. However, this binding capacity is usually saturable, owing to the saturation of the available binding sites in the proteins. To overcome this issue, some engineering processes envisage the loading of said binding proteins in order to increase the binding capacity of RBCs. In other cases, the strategy encompasses the engineering of enzymes so as to modify some key metabolites and, thus, modify oxygen release and/or other metabolites.

### 3.1 Inositol hexaphosphate and oxygen release

As stated above, hemoglobin is the major component of the RBCs and is responsible for oxygen delivery to all tissues of the body. Hemoglobin has a high affinity for oxygen that is physiologically affected by temperature, hydrogen ions, carbon dioxide, and intraerythrocyte 2,3-bisphosphoglycerate (2,3-BPG). Furthermore, a physiological change in hemoglobin affinity for oxygen favors oxygen binding in the lung and oxygen release to peripheral tissues. Based on these simple observations and other experiments, a decrease in O_2_ affinity was observed to enhance tissue O_2_ delivery (Lenfant et al., 1968). In attempts to produce erythrocytes with a lower oxygen affinity to be administered in a patient in need when normal blood flow is impaired, inositol hexaphosphate (InsP6)-loaded erythrocytes were prepared and investigated (Teisseire et al., 1985). InsP6 cannot diffuse through the erythrocytes’ membrane and is the most effective Hb allosteric effector (Benesch et al., 1977), even a thousand times more effective than 2,3-BPG. InsP6 was incorporated into erythrocytes by using a reversed osmotic-lysis process (Ropars et al., 1985) and shown to be effective in mini pig animal models and in sickle transgenic mice (Bourgeaux et al., 2012).

Although these and other data support the possible use of inositol hexaphosphate-loaded erythrocytes as effective treatments to improve the delivery of O_2_ to tissues in need, there is an ongoing debate about the advantages of higher or lower hemoglobin–oxygen affinity in humans, particularly during hypoxia (Webb et al., 2021) that require further investigations.

### 3.2 Engineering RBC metabolism and storage conditions

During their storage at refrigerated temperatures, red blood cells go through several biochemical and morphological modifications known as “storage lesion.” Indeed, during this period, 2,3-BPG and other high-energy phosphate compounds gradually decrease because of the reduced glycolytic activity at 1°C–6°C storage temperatures, the acidification of the intracellular pH, and the reversible and irreversible oxidation of the active site of rate-limiting glycolytic enzymes, such as glyceraldehyde 3-phosphate dehydrogenase.

2,3-Bisphosphoglycerate (2,3-BPG) is an intermediate metabolite present in high concentrations in RBCs (approximately 5 mM) and is the principal allosteric effector for hemoglobin (that is present at almost the same concentration as a tetramer), decreasing its affinity for oxygen. During storage conditions, the erythrocyte 2,3-BPG concentration decreases, which impacts the oxygen dissociation curve by shifting it to the left. This means that the oxygen delivery to tissues is reduced. In an attempt to overcome this problem, a number of additives have been proposed to extend 2,3-BPG preservation for weeks. Other authors have suggested that 2,3-BPG preservation should be obtained by modifying red cell metabolism through inhibition of key glycolytic enzymes (Vora, 1987; Beutler et al., 1995). The rationale for this approach is based on observations that patients who have pyruvate kinase deficiency have higher-than-normal levels of 2,3-BPG, while hexokinase-deficient patients have below-normal levels. Several years ago in our laboratory, we confirmed this assumption by loading human RBCs with hexokinase-inactivating antibodies (Magnani et al., 1989). RBCs loaded with these antibodies showed only 20% residual activity and lactate production was only 30% of controls, while the hexose monophosphate pathway was normal under basic conditions but only 12% of controls if the cell was stimulated by the addition of methylene blue. These cells were not able to maintain *in vitro* normal ATP and 2,3-BPG concentrations. In contrast, if RBCs were loaded with human hexokinase, they showed a doubled glycolytic activity, a normal hexose monophosphate pathway, and normal ATP and 2,3-BPG concentrations (Magnani et al., 1988). Storage at 4°C of these cells showed that 2,3-BPG was normal and ATP slightly decreased, proving that encapsulation of key glycolytic enzymes can provide a new way to maintain functionally active RBCs and prevent the loss of the most important hemoglobin effector (2,3-BPG) for several weeks (Magnani et al., 1989).

Furthermore, as the 2,3-BPG concentration decreases and hemoglobin affinity for O_2_ increases, there is also an accumulation of reactive species of oxygen (ROS) due to the impaired RBCs’ antioxidant capability (Yoshida et al., 2019). So, RBCs cannot face oxidative stress because of the insufficient glutathione synthesis and the inefficient pentose phosphate pathway to produce NADPH, which leads to the irreversible oxidation of key structural and functional proteins such as hemoglobin, band 3, and peroxiredoxin 2 (Bayer et al., 2015; Wither et al., 2016; Reisz et al., 2018). Some studies have exploited some new storage strategies to prevent RBC senescence, which may affect the transfusion efficiency and clinical implications including increased complications and mortality (Jones et al., 2019; Rogers et al., 2021). It was demonstrated that the supplementation of RBCs stored with uric acid and ascorbic acid, both antioxidant molecules, reduces oxidative damage and induces GSH synthesis (Tzounakas et al., 2022; Anastasiadi et al., 2023). Furthermore, washing stored RBCs with PBS seems to reduce senescence pathways, phosphatidylserine and band-3 exposure, and membrane fragility (Pulliam et al., 2021). D’Alessandro et al. proved that hypoxic storage conditions improved 2,3-BPG and GSH synthesis, and ameliorated redox metabolism (DʼAlessandro et al., 2020). Moreover, donor selection could influence the transfusion efficiency. For example, the enrollment of G6PD deficiency donors might result in increased hemolysis, mechanical fragility, and ROS accumulation after the transfusion, even if their adjustment to oxidative stress could make them better recipients of RBCs long-stored rather than donors (García-Roa et al., 2017; Putter and Seghatchian, 2017).

### 3.3 Immunophilin-loaded RBCs

**FIGURE 3:**
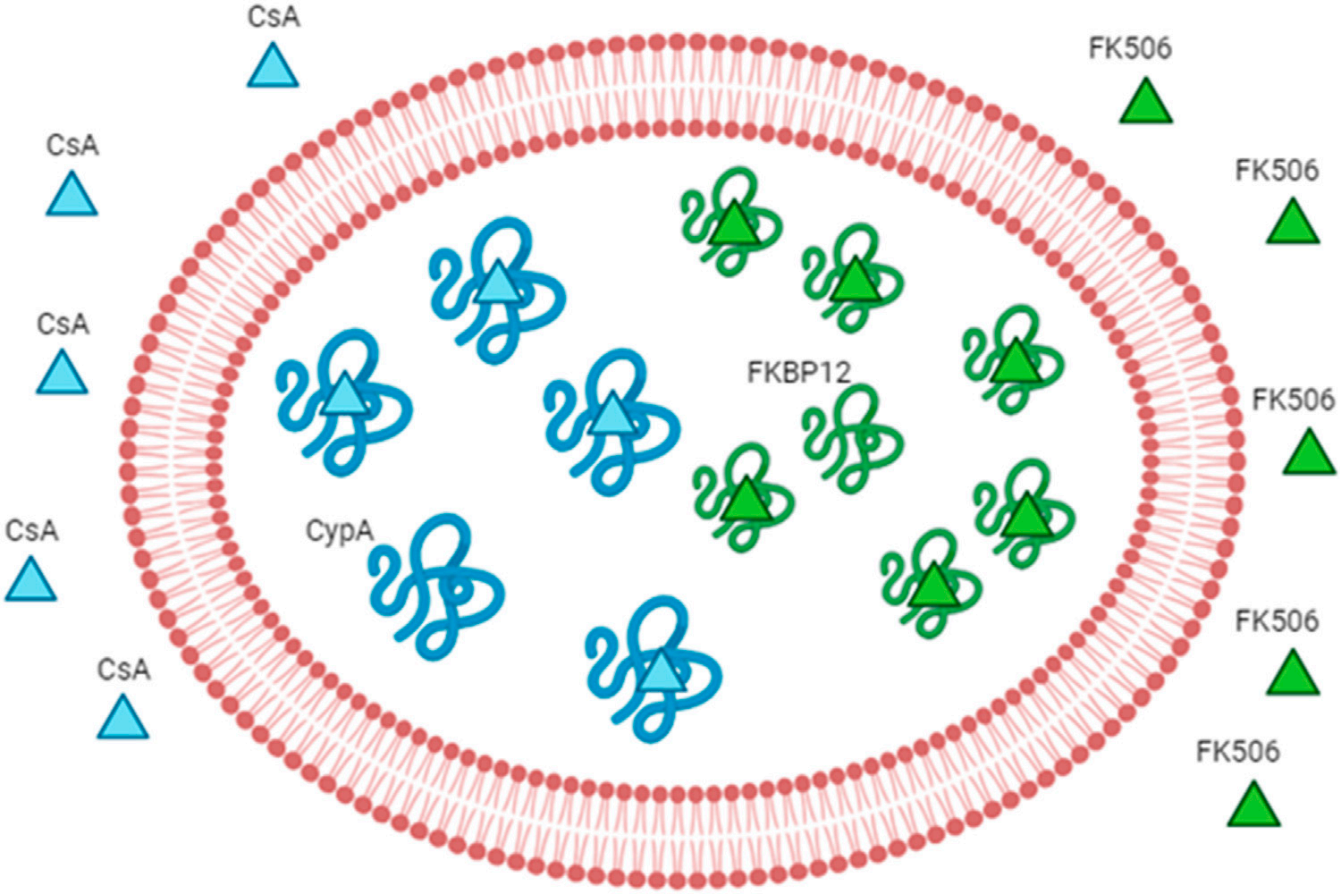
Engineered RBCs as immunophilin carriers. The figure schematizes how we can increase the drug-binding capacity of RBCs by increasing the concentration of the intracellular receptor (binding site). The example of immunosuppressants and immunophilins is given as a proof-of-concept. The immunophilin FKBP12 is known to bind to the immunosuppressant FK506 (also known as tacrolimus); thus, the overloading of the recombinant protein FKBP12 can increase the affinity for the same drug. The overloading of cyclophilin A can increase the affinity for cyclosporine A. Abbreviation in the figure: FKBP12, FK506-binding protein; CypA, cyclophilin A; CsA, cyclosporine A.

Biagiotti et al. first reported the pioneering use of engineered RBCs overloaded with cyclophilin A or FKBP12 as the drug delivery system for CsA or FKBP12, respectively (Biagiotti et al., 2011). As they can be spontaneously recruited into RBCs, our strategy is designed to increment the FK506 or CsA’s levels in RBCs by the augmentation of the cytosolic concentration of FKBP12 or cyclophilin A, respectively (Figure 3). In order to do that, recombinant FKBP12 and cyclophilin A were produced. Furthermore, a new approach is intended to enhance the FK506/CsA amount carried by erythrocytes. This involves recombinant forms of human FKBP12 and cyclophilin A loaded into RBCs through a process of hypotonic dialysis and isotonic resealing. Thus, engineered RBCs have the capacity to bind up to an order of magnitude more drugs than their native counterparts. In summary, our findings suggest that diffusible immunosuppressants could be sequestered into red blood cells by loading the respective target proteins. This proposes the potential use of immunophilin-loaded RBCs as a promising delivery system for immunosuppressive agents.

Moreover, a patent application has been filed by Magnani et al. (2010). The patent suggests two different uses of immunophilin-loaded RBCs: the first one proposes the loaded-RBC reinfusion into patients in need of immunosuppressive drugs after their *ex vivo* incubation with the drug of interest. In this way, the drug is not freely administrated, and the carriers slowly release the drug in circulation, which could eventually bind endogenous immunophilins. In contrast, according to the second modality, the patient will receive, first, the immunophilin-loaded red blood cells that can remain in circulation for months and then the drug (in any administration way). Thus, a significant portion of free drugs will be carried preferentially by the engineered cells due to their higher affinity. Both strategies are used in treating patients in need of immunosuppressive therapy, but which one is better in pharmacokinetics is still not known. However, the initial administration of immunophilin-loaded erythrocytes should coincide with the erythrocytes’ binding capacity saturation time with the selected drug. Once in circulation, the loaded erythrocytes will release the captured drug. Given their prolonged stay in circulation, they may also bind and transport other immunosuppressive drugs circulating in the system. In a further application, the patient’s RBCs could be loaded with more than one immunophilin simultaneously (i.e., FKBP12 and cyclophilin A), either separately or in combination with the same RBC. This approach aims to increase the quantity of immunosuppressive drugs administered to a patient without inducing pharmacological side effects.

The abovementioned example is potentially applicable to all human “druggable” proteins, that is, proteins that have been demonstrated to be able to bind a drug. The primary limiting step seems to lie in obtaining the recombinant forms of the selected proteins, produced under Good Manufacturing Practice (GMP) conditions and at a reasonable cost. According to the literature, single-protein domains or a combination thereof, can be exploited to create engineered RBCs to bind and transport all necessary drugs to a patient. Moreover, since the drug’s rate of dissociation from its protein-binding partner is determined by the protein’s affinity for the drug of choice, it may, one day, be possible to modify the specific site of the protein so that the drug would be released at a predetermined rate, maintaining the proper concentration of free drug in the bloodstream. Hence, by taking advantage of the potential erythrocytes loading with specific drug-binding proteins, then we will have new means to administer almost all drugs in patients.

### 3.4 RBC-mediated delivery of anti-tumor drugs

Anthracycline antibiotics are effective anti-cancer agents widely used for the treatment of different cancer diseases. Given that their high toxicity may provoke serious side effects, researchers are proposing different ways to reduce their toxicity without decreasing their efficacy. One of them is to extend the time of the drug in the bloodstream so that its dosage can be reduced. So, clinical studies evaluated the pharmacokinetics of RBC-bound daunorubicin and doxorubicin, in patients with acute leukemia and lymphoma, respectively. The results will be summarized in the following sections.

#### 3.4.1 Erythrocyte-bound daunorubicin

Pharmacokinetics of erythrocyte-bound daunorubicin (EBD) has been evaluated in 14 patients with acute leukemia, after the drug’s loading into RBCs (Skorokhod et al., 2004). Daunorubicin-loaded RBCs were prepared in order to have a final dose of 45 or 60 mg/m^2^ of the patient body surface, and the erythrocytes’ deformability was not affected during the EBD preparation. The results highlighted an increase in the average plasma and blood daunorubicin concentrations for the EBD compared to its standard free form, during the chemotherapy course. Moreover, EBD was better tolerated by patients, and in nine of them, side effects were uncommon, unlike those treated with the free form of daunorubicin. Although the primary purpose of this study was not to assess the antileukemic efficacy of EBD, it is worth mentioning that remission was achieved in eight out of 10 patients who received three infusions. Hence, the clinical application of daunorubicin-loaded red blood cells looks bright.

#### 3.4.2 Erythrocyte-bound doxorubicin

Intact RBCs can naturally bind anthracycline antibiotics in isotonic media (Kravtzoff et al., 1990; Ataullakhanov et al., 1992). Despite that, this binding is weak, so anthracycline antibiotics need to be immobilized by the use of glutaraldehyde (Ataullakhanov et al., 1994; Skorokhod et al., 2004). The use of engineered doxorubicin-loaded erythrocytes (DLEs) increased the drug’s presence in the bloodstream and facilitated its targeted delivery to the spleen, liver, and lungs, thereby reducing the doxorubicin load on other organs (Zocchi et al., 1989; Tonetti et al., 1990). Yet, glutaraldehyde treatment likely induced an unpredictable variation in antibiotic toxicity, and the DLE efficacy seems to be independent of the solidity of the binding between antibiotics and cells. Hence, DLE prepared without glutaraldehyde pharmacokinetics was evaluated in patients with lymphoproliferative disorders (Tonetti et al., 1992; Ataullakhanov et al., 1997). DLEs were set up to achieve a final dose of 25 or 50 mg/m^2^ post-infusion. The mixture was incubated at 37°C for 60 min and then infused into patients immediately thereafter. Compared to the standard administration of doxorubicin, the concentration over time of DLE administration was approximately five-fold higher for blood and six-fold higher for plasma (Ataullakhanov et al., 1997). Furthermore, DLE was well-tolerated in patients as neither prolonged or severe myelosuppression was observed nor evidence of cardiotoxicity was seen. Once more, these results refer to the delivery of anthracycline antibiotics through erythrocytes as a promising tumor treatment.

More recently, Lucas et al. submitted a methodology for the synthesis of DOX-loaded red blood cells (RBC-DOX) through electrophoresis (Lucas et al., 2019). The strategy has been tested both in *in vitro* models and in preclinical models, manifesting the faculty of RBCs to carry higher doses of DOX and limit cardiac toxicity with superior antitumorigenic effects.

### 3.5 Conclusion

In conclusion, human RBCs are not only hemoglobin-containing bags involved in gas exchanges but may have great importance in drug transport. This opens the way to two fields of investigation. On one hand, being that RBC partitioning has an enormous impact on drug pharmacokinetics, clinicians must take into account the therapeutic drug monitoring in whole blood for those drugs showing a high blood-to-plasma ratio. On the other hand, biomedical researchers can set up innovative strategies that are aimed at increasing the RBC power to bind and transport selected drugs by exploiting some of their natural features.
