# Corrigendum: Drug transport by red blood cells

**DOI:** 10.3389/fphys.2024.1454770

**Published:** 2024-07-30

**Authors:** Sara Biagiotti, Elena Perla, Mauro Magnani

**Affiliations:** Department of Biomolecular Sciences, University of Urbino Carlo Bo, Urbino, Italy

**Keywords:** red blood cells, drug transport, blood-to-plasma ratio, pharmacokinetic, blood distribution

In the published article, an **Author** name was incorrectly written as Elena Pirla. The correct spelling is Elena Perla.

The authors apologize for this error and state that this does not change the scientific conclusions of the article in any way. The original article has been updated.

